# Wild *Anopheles funestus* Mosquito Genotypes Are Permissive for Infection with the Rodent Malaria Parasite, *Plasmodium berghei*

**DOI:** 10.1371/journal.pone.0061181

**Published:** 2013-04-08

**Authors:** Jiannong Xu, Julián F. Hillyer, Boubacar Coulibaly, Madjou Sacko, Adama Dao, Oumou Niaré, Michelle M. Riehle, Sekou F. Traoré, Kenneth D. Vernick

**Affiliations:** 1 Unit of Insect Vector Genetics and Genomics, Department of Parasitology and Mycology, Institut Pasteur, Paris, France; 2 Microbial and Plant Genomics Institute, Department of Microbiology, University of Minnesota, Saint Paul, Minnesota, United States of America; 3 Department of Biology, New Mexico State University, Las Cruces, New Mexico, United States of America; 4 Department of Biological Sciences and Institute for Global Health, Vanderbilt University, Nashville, Tennessee, United States of America; 5 Malaria Research and Training Center, University of Bamako, Bamako, Mali; Kansas State University, United States of America

## Abstract

**Background:**

Malaria parasites undergo complex developmental transitions within the mosquito vector. A commonly used laboratory model for studies of mosquito-malaria interaction is the rodent parasite, *P. berghei*. *Anopheles funestus* is a major malaria vector in sub-Saharan Africa but has received less attention than the sympatric species, *Anopheles gambiae*. The imminent completion of the *A. funestus* genome sequence will provide currently lacking molecular tools to describe malaria parasite interactions in this mosquito, but previous reports suggested that *A. funestus* is not permissive for *P. berghei* development.

**Methods:**

An *A. funestus* population was generated in the laboratory by capturing female wild mosquitoes in Mali, allowing them to oviposit, and rearing the eggs to adults. These F1 progeny of wild mosquitoes were allowed to feed on mice infected with a fluorescent *P. berghei* strain. Fluorescence microscopy was used to track parasite development inside the mosquito, salivary gland sporozoites were tested for infectivity to mice, and parasite development in *A. funestus* was compared to *A. gambiae*.

**Results:**

*P. berghei* oocysts were detectable on *A. funestus* midguts by 7 days post-infection. By 18–20 days post-infection, sporozoites had invaded the median and distal lateral lobes of the salivary glands, and hemocoel sporozoites were observed in the hemolymph. Mosquitoes were capable of infecting mice via bite, demonstrating that *A. funestus* supports the complete life cycle of *P. berghei*. In a random sample of wild mosquito genotypes, *A. funestus* prevalence of infection and the characteristics of parasite development were similar to that observed in *A. gambiae*-*P. berghei* infections.

**Conclusions:**

The data presented in this study establish an experimental laboratory model for *Plasmodium* infection of *A. funestus*, an important vector of human malaria. Studying *A. funestus*-*Plasmodium* interactions is now feasible in a laboratory setting. This information lays the groundwork for exploitation of the awaited genome sequence of *A. funestus*.

## Introduction

Close to half of the world's population is at risk of malaria infection [1], [2]. Attempts to curtail disease transmission have focused on the development of drugs to treat infected individuals, insecticide spraying to kill the mosquito vectors, and the use of physical barriers to prevent vector-human contact. Understanding the biology of mosquito-malaria interactions may aid in the development of a new generation of vector-based measures for malaria control.

In sub-Saharan Africa, where greater than 85% of malaria-associated mortality occurs [1], the major *Plasmodium* vectors are *A. gambiae*, *A. arabiensis*, *A. funestus*, *A. nili*, and *A. moucheti*, with heterogeneities in populations occurring both geographically and seasonally [3], [4]. To date, due to practical reasons such as extensive genome information and relative ease of colonization, most studies on mosquito-*Plasmodium* interactions have focused on the African mosquito, *A. gambiae*, and the Asian mosquito, *A. stephensi*. However, *A. funestus* is a major malaria vector in certain regions of Africa [3], plays a more prominent role than *A. gambiae* during the dry season [5], and in areas where it co-exists with *A. gambiae* it has been observed to have higher infection rates [6]. Thus, understanding the vector biology of *A. funestus* needs to be part of successful malaria control, but currently little is known about this species. Towards this end, we and colleagues recently determined and reported the complete transcriptome sequence of *A. funestus* using RNA-seq next-generation sequencing technology [7].

When studying mosquito-malaria interactions, model parasite systems are often used because of the ease of manipulation and the laboratory safety afforded by using parasites incapable of infecting humans. One model parasite commonly used is *P. berghei*, a rodent malaria species originally isolated from the salivary glands of *A. dureni* and whose vertebrate host in nature is the Central African tree rat, *Thamnomys surdaster* [8], [9]. *P. berghei* can be genetically manipulated, and marked transgenic parasites are easily visualized in mosquito and mammalian tissues [10]–[14]. To date, experimental infections have shown that *P. berghei* is capable of completing its life cycle in *A. quadrimaculatus*, *A. freeborni*, *A. stephensi*, *A. annupiles*, *A. atroparvus*, *A. maculipennis*, and *A. gambiae* [15]–[21]. *A. albimanus*, a major malaria vector in South America, is not an efficient laboratory vector of *P. berghei* [19], [22], and the African mosquito *A. quadriannulatus* Species A, a member of the *A. gambiae* species complex, also is not permissive for *P. berghei* development [23]. Ten other anopheline species have been described as being resistant to *P. berghei*, including the only published report using *A. funestus*, which states that *P. berghei* is unable to infect *A. funestus* [18], [20]. Here, we show for the first time that *P. berghei* can indeed infect and complete its life cycle in random natural genotypes of the important African malaria vector *A. funestus*, and that infection progression proceeds with similar kinetics and efficiency to that observed in *A. gambiae*.

## Methods

### Mosquitoes

Wild *A. funestus* females were collected indoors in the village of Niono, Mali, West Africa. No specific permits were required for the collection of mosquitoes. Mosquitoes were collected inside village houses by agreement of the residents. Because they were captured resting indoors, the captured females, morphologically identified as *A. funestus sensu lato*, had already mated and taken a bloodmeal in nature. Mosquitoes were housed in an environmental chamber at 26°C and 75% relative humidity. For each experiment approximately 50 wild-fed gravid females were allowed to oviposit collectively. The resulting eggs were grown to adult mosquitoes under standard insectary conditions, including larval food, as used for *A. gambiae* [12].

### Parasites and infection

To determine the permissiveness of *A. funestus* to *P. berghei* infection, approximately 200 5–7 day old adult females (raised from wild larvae) were starved overnight and allowed to feed for 30 min on Swiss Webster mice with a parasitemia of approximately 10% and a gametocytemia of approximately 2%. Following blood feeding, unfed mosquitoes were removed and fed mosquitoes were maintained at 20.5°C and 75% relative humidity. Infections were done using the PbGFP_CON_ transgenic strain of *P. berghei* that constitutively expresses green fluorescent protein (GFP) [10].

This study was carried out in strict accordance with the recommendations in the Guide for the Care and Use of Laboratory Animals of the National Institutes of Health. The protocol was approved by the Institutional Animal Care and Use Committee of the University of Minnesota (Permit Number: 1201A08951, NIH Animal Welfare Assurance number: A3456). All mosquito infections were performed by feeding on mice anesthetized with ketamine/xylazine, and all efforts were made to minimize suffering.

### Parasite quantitation and statistical analysis

To qualitatively and quantitatively assess the permissiveness of *A. funestus* to *P. berghei*, intact mosquitoes and dissected mosquito tissues were examined at 7 days post-feeding to assay midgut stages of infection and at 18–20 days post-feeding to assay sporozoite migration and salivary gland invasion. Oocyst infection prevalence is defined as the percentage of mosquitoes that become infected with oocysts, and oocyst intensity is the mean and median number of oocysts in the midguts of infected mosquitoes [24].

The data presented in this study represent the results from two independent field collections and laboratory experiments. For comparison, parallel infections were carried out by feeding mosquitoes of the sympatric species, *A. gambiae* (G3 colony) on the same *P. berghei*-infected mice. *A. gambiae* were reared and maintained under the same conditions described for *A. funestus*.

### Cell imaging

Visual observations were done under bright field illumination, differential-interference-contrast (DIC), and GFP epi-fluorescence using a Nikon Eclipse E600 upright microscope connected to a CoolSNAPES digital camera (Photometrics, Tucson, AZ). Digital images were taken using MetaVue Imaging Software (Universal Imaging Corporation, Downingtown, PA), bright field images HiGauss filtered using Image-Pro Plus (Media Cybernetics, Silver Spring, MD), and histogram stretches, stitching, and overlays done using Adobe Photoshop (Adobe Systems, San Jose, CA).

## Results and Discussion

We used the F1 progeny of field-collected *A. funestus* females to assess whether *P. berghei* can complete its life cycle in this mosquito species, and we did not attempt to obtain further generations. Thus, the mosquitoes tested should represent an unbiased sample of random wild mosquito genotypes.

**Figure 1:**
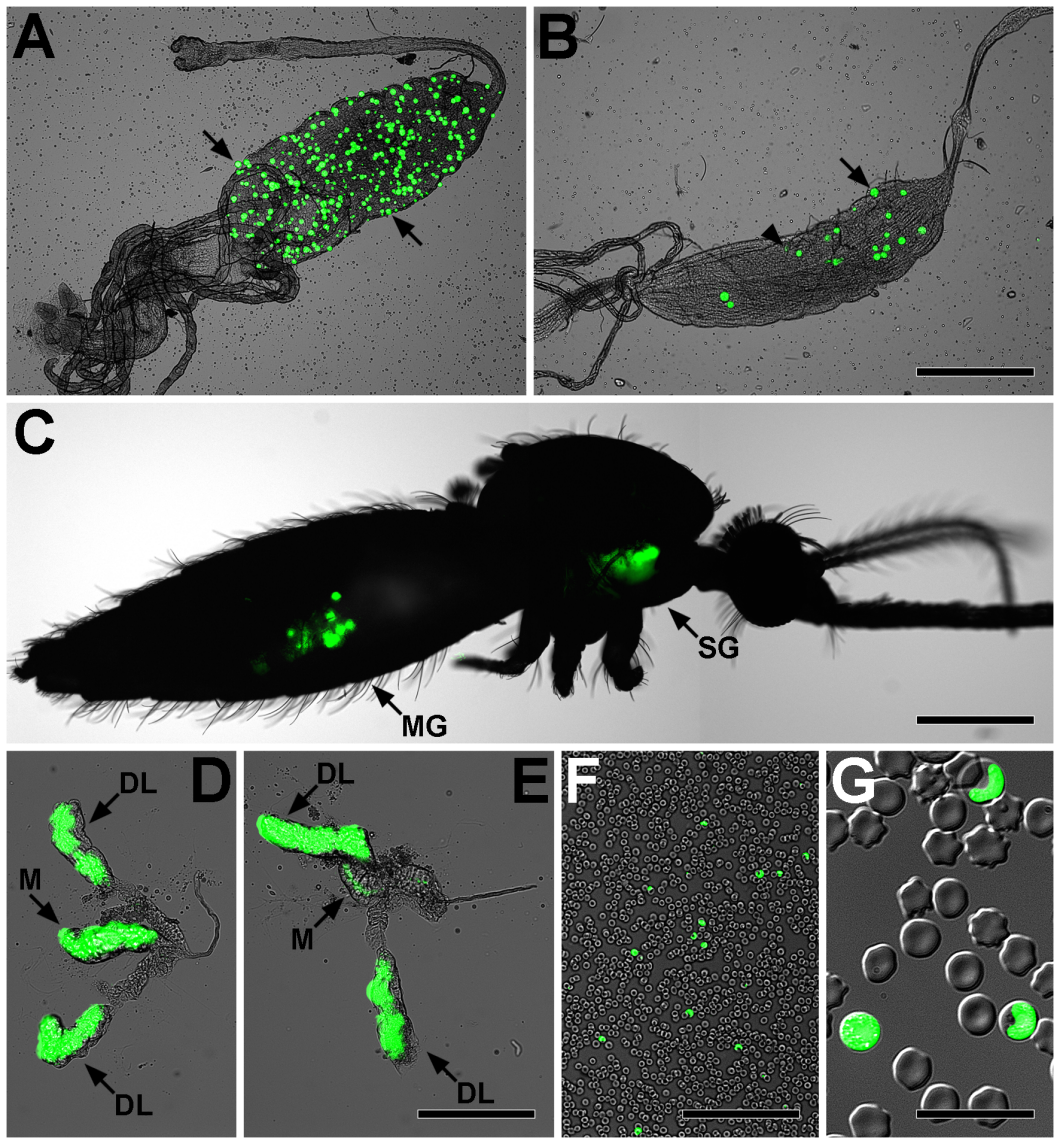
Light and GFP epi-fluorescence microscopy show fluorescent *Plasmodium berghei* parasites developing in *Anopheles funestus* mosquitoes and murine erythrocytes. **A.** *A. funestus* midgut with greater than 300 *P. berghei* oocysts (e.g., arrows) at 7 days post-infection. **B.** *A. funestus* midgut showing normal oocysts (e.g., arrow) and oocysts that have recently undergone rupture (e.g., arrowhead) at 20 days post-infection. **C.** Imaging of fluorescent parasites through the cuticle of a live *A. funestus* showing parasite development in the midgut (MG) and salivary glands (SG). Note that tissues presented in panels B, D, and E originated from this mosquito. **D–E.** *A. funestus* salivary glands showing that sporozoites preferentially invade the median (M) and distal lateral (DL) lobes. **F–G.** Blood smear from a mouse exposed to *P. berghei* via mosquito bite showing infected erythrocytes, indicating that *A. funestus* salivary gland sporozoites are infective to the vertebrate host and that the parasite can complete its life cycle inside the insect vector. Bars: A–C = 500 µm; D–E = 200 µm; F = 100 µm; G = 20 µm.

Following blood feeding on infected mice, numerous *P. berghei* oocysts were observed on *A. funestus* midguts as early as 7 days post-feeding, the earliest time point tested (Figure 1A). By 18–20 days post-infection, both midgut oocysts and salivary gland sporozoites were observed in dissected tissues (Figure 1B, D–E), and through the cuticle of live mosquitoes (Figure 1C). By this time several oocysts had ruptured, and 57% (9/17) of infected mosquitoes carried salivary gland sporozoites. In the hemocoel, migrating sporozoites were observed to flow with the hemolymph through the dorsal vessel in a manner similar to that recently reported in the *A. gambiae*-*P. berghei* system [12], [14]. Furthermore, sporozoites were also occasionally observed attached to the cuticle and in the appendages. When the salivary glands were closely examined, sporozoites were observed to preferentially invade the median and distal lateral lobes (Figure 1D–E). These observations are in agreement with reports in other mosquito species [25], [26], including the *A. gambiae*-*P. berghei* system [27].

To test the infectivity of *A. funestus* salivary gland sporozoites to mice, 15 infected mosquitoes (19 days post-*P. berghei* infection) were fed on two normal mice. One mouse became infected as determined by the visualization of blood stage parasites at 7 days post-mosquito bite (Figure 1F–G). These experiments indicate that *P. berghei* is capable of completing its life cycle in *A. funestus*.

**Figure 2:**
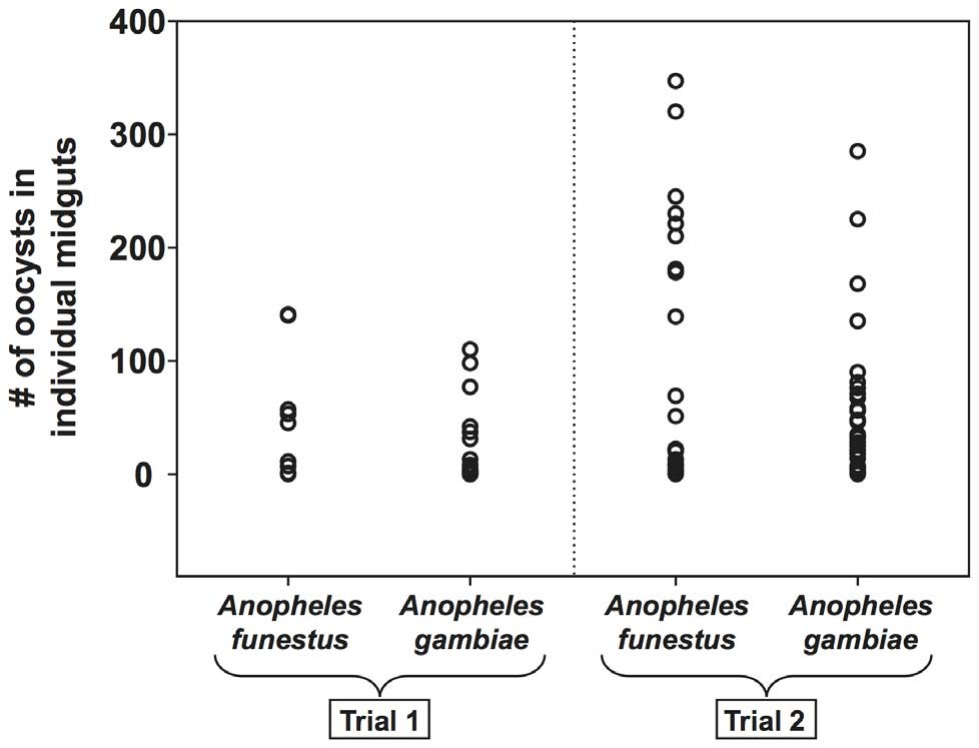
*Anopheles funestus* and *Anopheles gambiae* mosquitoes display equivalent susceptibility to *Plasmodium berghei* infection. *A. gambiae* and *A. funestus* mosquitoes were fed on the same infected *P. berghei*-infected mouse in two replicate experiments, and midgut oocyst infections were quantified at 7 days post-infection. Each circle represents the number of midgut oocysts in an individual mosquito.

To compare parasite development in *A. funestus* versus *A. gambiae*, parallel infections were tracked for the first 7 days post-infection (Figure 2). In the first trial, *A. funestus* prevalence of infection, as determined by the presence of fluorescent *P. berghei* oocysts on the midguts of individual mosquitoes, was 62.5% (10/16) and median oocyst intensity was 28 (mean = 47; range = 1–141). In a parallel infection using *A. gambiae*, prevalence of infection was 56% (20/36) and median oocyst intensity was 4 (mean = 23; range = 1–110). The second trial yielded a similar comparison: *A. funestus* prevalence of infection was 95% (19/20) as compared to 97% (29/30) for *A. gambiae*, and median oocyst intensity was 69 (mean = 120; range = 1–347) for *A. funestus* and 35 (mean = 56; range = 1–285) for *A. gambiae*. Overall, *P. berghei* infection prevalence was equivalent in the two mosquito species. Although both trials showed a trend for higher oocyst intensities in *A. funestus*, the difference was not statistically significant (Mann-Whitney *P*>0.05). However, due to the negative binomial distribution of oocysts in infected midguts [28], [29], large sample sizes would be necessary to yield statistically significant estimates of mean infection intensity.

## Conclusions

This study demonstrates that *P. berghei* can complete its life cycle in *A. funestus*. Several hurdles remain to be overcome before unraveling the molecular interactions between *Plasmodium* parasites and *A. funestus*, including the lack of available diverse and robust laboratory colonies. Nevertheless, the data presented in this study, together with the understanding that *A. funestus* is a major vector of human malaria, illustrate that, once the reference genome sequence of *A. funestus* is available, studying *A. funestus*-*Plasmodium* interactions is feasible and warranted in a laboratory setting.
